# Pirfenidone suppressed triple‐negative breast cancer metastasis by inhibiting the activity of the TGF‐β/SMAD pathway

**DOI:** 10.1111/jcmm.17673

**Published:** 2023-01-18

**Authors:** Daiqin Luo, Xianlin Zeng, Shuling Zhang, Daohong Li, Zhimei Cheng, Yun Wang, Jinhua Long, Zuquan Hu, Shiqi Long, Jing Zhou, Shuai Zhang, Zhu Zeng

**Affiliations:** ^1^ School of Basic Medical Sciences/School of Biology & Engineering Guizhou Medical University Guiyang China; ^2^ Engineering Center of cellular immunotherapy of Guizhou Province Guiyang China; ^3^ Department of oncology Affiliated Hospital of Guizhou Medical University Guiyang China; ^4^ Department of oncology Affiliated Cancer Hospital of Guizhou Medical University Guiyang China; ^5^ Key Laboratory of infectious immunity and antibody engineering of Guizhou Province Guiyang China; ^6^ School of Public Health Guizhou Medical University Guiyang China; ^7^ Department of Interventional Radiology Affiliated Hospital of Guizhou Medical University Guiyang China; ^8^ Key Laboratory of Endemic and Ethnic Diseases, Ministry of Education Guizhou Medical University Guiyang China; ^9^ State Key Laboratory of Functions & Applications of Medicinal Plants Guizhou Medical University Guiyang China

**Keywords:** breast cancer, EMT, metastasis, Pirfenidone, TGF‐β

## Abstract

Among breast cancer patients, metastases are the leading cause of death. Despite decades of effort, little progress has been made to improve the treatment of breast cancer metastases, especially triple‐negative breast cancer (TNBC). The extracellular matrix plays an important role in tumour growth and metastasis by causing its deposition, remodelling, and signalling. As we know, the process of fibrosis results in excessive amounts of extracellular matrix being deposited within the cells. So, it will be interesting to study if the use of anti‐fibrotic drugs in combination with conventional chemotherapy drugs can produce synergistic antitumor effects. In this study, we assessed the efficacy of Pirfenidone (PFD), an FDA‐approved medication for the treatment of idiopathic pulmonary fibrosis, on TNBC cells as well as its anti‐tumour effects in xenograft tumour model. PFD inhibited in a dose‐dependent manner breast cancer cell proliferation, migration, and invasion, while promoted their apoptosis in vitro. PFD also suppressed TGF‐β‐induced activation of Smad signalling pathway and expression level of EMT‐inducing transcription factors (e.g. SNAI2, TWIST1, ZEB1) as well as the mesenchymal genes such as VIMENTIN and N‐Cadherin. On the contrary, the expression level of epithelial marker gene E‐Cadherin was up‐regulated in the presence of PFD. In vivo, PFD alone exerted a milder but significant anti‐tumour effect than the chemotherapy drug nanoparticle albumin‐bound paclitaxel (nab‐PTX) did in the breast cancer xenograft mouse model. Interestingly, PFD synergistically boosted the cancer‐killing effect of nab‐PTX. Furthermore, Our data suggest that PFD suppressed breast cancer metastasis by inhibiting the activity of the TGFβ/SMAD pathway.

## 1 INTRODUCTION

Every year, there are about 1 million women worldwide are diagnosed with breast cancer, among which 15%–20% patients are estimated to be the triple‐negative phenotype.^1^, ^2^ Triple‐negative breast cancer (TNBC) carries a high risk of early metastasis and has a poorer prognosis than other breast cancer subtypes.^3^ Therefore, one of the major goals of breast cancer research is to prevent metastasis of breast tumours. Therefore, TNBC patients are unlikely to benefit greatly from current therapies, and new therapies are urgently needed. Additionally, it is not easy to treat TNBC due to the chemo‐resistant nature of TNBC. Therefore, exploring the underlying mechanisms of TNBC progression is needed for novel therapies.

Breast tumours are usually detected by manual palpation because they are more rigid than the surrounding normal tissue. This increased in tissue stiffness or matrix stiffness plays an important role in tumour progression. Organized collagen fibre arrangement is a surrogate marker for increasing matrix stiffness in the tumour microenvironment and is associated with breast tumour progression.^4^, ^5^, ^6^ On a stiffened matrix similar to breast tumours, cells lose apical‐base polarity, form weaker junctions, and invade through the basement membrane.^7^

It's well known that tumour microenvironment influences tumorigenesis, cancer progression, and metastasis.^8^, ^9^ Because the metabolisms of tumour cells are more vigorous than normal cells, during the progression, breast cancer is characterized by stiffening of the cell‐extracellular matrix (ECM) due to excessive deposition and cross‐linking of collagen, which greatly affects tumour behaviour and fate.^10^ As cancer progresses, intercellular communication and ECM interactions are closely linked to the development of metastatic and localized tumour microenvironments (TMEs) that allow metastasis to occur.^11^ Breast cancer development is supported by increased collagen deposition, which provides both physical and biochemical signals that promote tumour growth and invasion.^12^ The increased extracellular matrix stiffness of breast cancer promotes epithelial to mesenchymal cell transformation, leading to cell infiltration and metastasis.^13^ From a biomechanics and mechanobiology perspective, there is a strong correlation between stiffening of the TME and activation of epithelial‐to‐mesenchymal transition pathways, tumour growth, and increased malignancy.^14^, ^15^, ^16^, ^17^ Therefore, it is crucial to understand whether the progression and metastasis of breast cancer can be regulated by reducing the degree of extracellular matrix fibrosis.

Pirfenidone (PFD), a pyridone derivative with anti‐fibrotic and anti‐inflammatory properties, was discovered and extensively tested for the treatment of lung fibrosis.^18^ Since the process of metastasis is accompanied by the fibrosis of tumour tissue, so if reversing the degree of tumour tissue fibrosis can reverse tumour metastasis is deserved consideration. The mechanism of PFD is partially due to its inhibition on production/activity of transforming growth factor β (TGF‐β).^19^, ^20^ On one hand, TGF‐β is such a potent driver of the transition that it has been frequently used as a positive control in epithelial‐mesenchymal transition (EMT) ‐inducing studies.^21^ On the other hand, EMT is a developmentally conserved biological process by which a polarized epithelial cell loses its attachment on basement membrane while acquiring capacities of migration and invasion. As a result of the reprograming, the epithelial cell is endowed with a mesenchymal phenotype.^22^, ^23^ In addition to its essential contribution to embryonic development and tissue repair, evidence shows that EMT is also involved in tumorigenesis.^24^, ^25^ Tumour cells undergone EMT acquire the properties of invasion, infiltrate into the surrounding stroma and form a microenvironment facilitating tumour growth and metastasis.^26^ The process also directly confers cancer cells apoptosis‐resisting property to chemotherapy and drugs.^27^ In view of the strong EMT‐inducing effect of TGF‐β, a drug suppressing TGF‐β signalling pathway could be a potential strategy treating EMT‐caused metastasis in breast cancer.

Here, we hypothesized that the combination of anti‐fibrotic drug with commonly adopted chemotherapy may produce synergistic effects. Therefore, we aimed to investigate the efficacy and safety of PFD combined with nab‐PTX as the treatment of TNBC. The results showed that the expression of TGF‐β is overall higher in breast cancer tissues than that of in healthy controls and negatively associated with disease free survival (DSF) of the disease. In vitro, experiments showed that PFD induced breast cancer cell MDA‐MB‐231 apoptosis and inhibited their proliferation, migration, and invasion. These breast cancer prevention effects of PFD were partially attributed to its dose‐dependent suppression on both TGF‐β expression and TGF‐β‐stimulated Smad signalling pathways. Such anti‐cancer function of PFD was further validated in vivo in mouse xenograft breast cancer model. The results from our study highlighted the potential medical value of PFD as a suppressor of EMT‐mediated breast cancer metastasis.

## 2 MATERIALS AND METHODS

### 2.1 Materials

Pirfenidone was purchased from Sigma Aldrich and resuspended in DMSO per manufacturers recommendation. Type‐I and type‐II TGF‐β receptors antagonist LY2109761 were purchased from Med Chem Express. PTX nanoparticle for injection (albumin bound) (Aiyue®) was produced by Jiangsu Hengrui Pharmaceutical Co., Ltd. Fetal bovine serum (FBS) was purchased from ExCell Biotech Co., Ltd.Matrigel® was obtained from Corning.

### 2.2 Cell culture and treatments

Breast cancer cell lines MDA‐MB‐231 were purchased from the Chinese Academy of Sciences, and cultured in L15 (Zhong Qiao Xin Zhou) medium supplemented with 10% Fetal Bovine Serum (FBS) and penicillin (100 IU/ml) /streptomycin (100 μg/ml) (Gibco) in a humidified incubator at 37°C. For testing the effect of PFD on TGF‐β‐induced activation of Smad signalling pathway and expression of EMT‐related genes, the breast cancer cells were pre‐treated with 5 ng/ml TGF‐β (Millipore Sigma) for 24 h, followed by addition of PFD (4 mM) or LY2109761 (2 μM) for another 24 h.

### 2.3 Cell viability assay

Cell viability of the breast cancer cells in the presence of gradient PFD was determined using the Cell Counting Kit 8 (CCK‐8) (TargetMol) according to the manufacturer's protocol. Briefly, MDA‐MB‐231 cells were seeded in 96‐well plates at a density of 3000 cells per well in 100 μl growth medium and allowed to attach for 24 h before the treatment. An equal volume of growth medium containing PFD at concentrations between 0.2 and 20 mM was then added to the corresponding wells, giving final PFD concentrations between 0.1 and 10 mM. Twenty‐four hours after the treatment, 10 μl CCK‐8 solution was added to each well. The plate was protected from light and incubated in 37°C incubator for another 1 h. The absorbance was measured at 450 nm.

### 2.4 Clonogenic assay

MDA‐MB‐231 cells were plated at a low‐density of 800 cells per well of 6‐well dish and grown in growth medium containing gradient PFD for 14 days. The cells were then fixed with 4% paraformaldehyde and stained with 0.05% crystal violet solution (Sigma‐Aldrich). The colonies were imaged and counted under an inverted microscope.

### 2.5 Apoptosis analysis by flow cytometry

Apoptosis was determined using FITC‐Annexin V/PI double staining kit (meilunbio) by flow cytometry. In brief, MDA‐MB‐231 cells in 6‐well plate were cultured in the absence or presence of gradient PFD for 24 h. All the cells including the floating and attached cells were collected by brief centrifugation and resuspended in 100 μl binding buffer containing 5 μl Annexin V and 5 μl PI. After 15 min incubation at room temperature in the dark, cells were resuspended in 400 μl binding buffer and cells were analysed via a flow cytometer (BD FACSCanto™ Flow Cytometer) and FlowJo software version 10. FITC‐labelled Annexin V‐positive cells were determined as apoptotic cells.

### 2.6 Wound healing assay

For assessing cell migrating capacity, MDA‐MB‐231 cells were grown in 6‐well plates until confluent. A scratch across the centre of each well was created using a 200 μl pipette tip. The disturbed cells were gently washed away with PBS, while the remaining cells were treated with gradient PFD in serum‐free growth medium for 24, 48, and 72 h, respectively. Images were taken at 0 and corresponding endpoints after the scratch. The migration rate (%) = (Distance_0h_ – Distance_24h_) /Distance_0h_ × 100%.

### 2.7 Transwell assay

For comparing cell migration, MDA‐MB‐231 cells were pre‐treated with gradient PFD for 24 h followed by seeding onto the upper chamber of the 24‐well BD Migration Chambers (BD Bioscience) at a density of 5 _×_ 10^5^ cells/well with L15 containing 0.5% FBS. The cells were allowed to invade through the 8‐mum pore size filter membrane and attach on the bottom of the well filled with complete growth medium for 24 h. The migrated cells were then fixed with 4% paraformaldehyde and stained with 0.05% crystal violet solution (Sigma‐Aldrich). The colonies were imaged and counted under an inverted microscope. For assessing cell invasion, the upper insert chambers were pre‐coated with Matrigel (300 μg/ml, 100 μl per chamber) (BD Biosciences) followed by performing the afore‐described procedure for the migration assay.

### 2.8 Western blot assay

Total protein from breast cancer cells and tumours were extracted using RIPA cell lysis solution (Solarbio) supplemented with protease inhibitor cocktail (Sigma) and quantified using BCA protein assay kit. Twenty micrograms of total protein from each sample were separated on SDS‐PAGE gel and transferred onto 0.45‐μm nitrocellulose membrane (Millipore). Primary antibodies used were anti‐Smad2 (1:2000; Abcam, AB40855), anti‐Smad3 (1:1000; Abcam, AB40854), anti‐Smad4 (1:5000; Abcam, AB40759), anti‐phospho‐Smad2 (1:2000; GeneTex, GTX03203), anti‐phospho‐Smad3 (1:2000; GeneTex, GTX00969), anti‐β‐ACTIN (1:10,000; proteintech，Cat No:66009), anti‐N‐cadherin (1:5000; Abcam, AB76011); anti‐E‐Cadherin (1:1000; Abcam, AB181296), anti‐Vimentin (1:2000; Abcam, AB16700) and anti‐TGF‐β (1:1000; proteintech, Cat No:21898). The immunolabelled proteins were detected by ECL detection system. Quantification of the protein bands were performed using ImageJ software (https://imagej.nih.gov/ij).

### 2.9 Total RNA extraction and quantitative Real‐Time PCR

total RNA from breast cancer cells was isolated using TRIzol™ Reagent (Life Technologies) according to the manual. Single‐stranded cDNA was synthesized using a Fastquant reverse kit (Tiangen). Quantitative RT‐PCR analysis was performed using the SYBR Green (TaKaLa) on Bio‐Rad CFX qPCR instrument. The primers used were listed in Table 1.

**TABLE 1:** Primers used for qRT‐PCR in this study.

Primer ID	Sequence (5′‐3′)
CDH1‐F	AGTCACTGACACCAACGATAAT
CDH1‐R	ATCGTTGTTCACTGGATTTGTG
CDH2‐F	CGATAAGGATCAACCCCATACA
CDH2‐R	TTCAAAGTCGATTGGTTTGACC
KRT8‐F	TACATGAACAAGGTAGAGCTGG
KRT8‐R	CCGGATCTCCTCTTCATATAGC
TJP1‐F	AAAGAGAAAGGTGAAACACTGC
TJP1‐R	TTTTAGAGCAAAAGACCAACCG
FN1‐F	AATAGATGCAACGATCAGGACA
FN1‐R	GCAGGTTTCCTCGATTATCCTT
β‐Actin‐F	TGACGGGGTCACCCACACTGTGCC
β‐Actin‐R	TAGAAGCATTTGCGGTGGACGATG
MMP2‐F	ATTGTATTTGATGGCATCGCTC
MMP2‐R	ATTCATTCCCTGCAAAGAACAC
SNAI1‐F	CCTCGCTGCCAATGCTCATCTG
SNAI1‐R	AGCCTTTCCCACTGTCCTCATCTG
SNAI2‐F	CTGTGACAAGGAATATGTGAGC
SNAI2‐R	CTAATGTGTCCTTGAAGCAACC
TWIST1‐F	GTACATCGACTTCCTCTACCAG
TWIST1‐R	CATCCTCCAGACCGAGAAG
ZEB1‐F	CAGGCAAAGTAAATATCCCTGC
ZEB1‐R	GGTAAAACTGGGGAGTTAGTCA
VIM‐F	CCTTCGTGAATACCAAGACCTGCTC
VIM‐R	AATCCTGCTCTCCTCGCCTTCC

### 2.10 Immunofluorescence

MDA‐MB‐231 cells were fixed by 4% paraformaldehyde and permeabilized with 0.5% Triton X‐100. After blocking for non‐specific binding sites with 5% BSA, the cells were stained with anti‐TGF‐β primary antibody (1:200; proteintech, Cat No:21898) overnight at 4°C. On next day, cells were washed with PBS, and the primary antibody was removed, followed by fluorescence secondary antibody. The nucleus was stained with 4′, 6‐diamidino‐2‐phenylindole (DAPI), and an anti‐fluorescence quenching agent (Solarbio).

### 2.11 Immunohistochemistry

Tumours were fixed with 4% paraformaldehyde and embedded with paraffin followed by sectioned into 5 μm sections. The sections were deparaffinized, rehydrated, and heated in citrate buffers of pH 6 for antigen retrieval. After blocking with normal goat serum, the sections were incubated overnight with primary antibodies against E‐Cadherin (1:30; Abcam, AB181296), Vimentin (1:200; Abcam, AB16700) and TGF‐β (1:200; proteintech, Cat No:21898), respectively, at 4°C. HPR‐conjugated secondary antibody (Thermo Fisher Scientific) was added to the PBST‐washed sections for a 1 h‐incubation at room temperature. The sections were then stained with diaminobenzidine (DAB) and counterstained with haematoxylin for visualization and imaging under a digital microscope.

### 2.12 Enzyme‐linked immunosorbent assay (ELISA)

Collagen Iα, Collagen III, and TGF‐β1 were measured using a double antibody sandwich avidin–biotin complex ELISA. To measure the concentrations, ELISA kits for Collagen Iα, Collagen III, and TGF‐β1(Elabscience, Houston) were used according to the manufacturer's instructions. The absorbance values of the samples were measured at 450 nm using an ELISA plate reader. The Collagen Iα, Collagen III, and TGF‐β1 concentration was positively proportionate to the absorbance values; therefore, the Collagen Iα, Collagen III, and TGF‐β1 concentrations in the samples were calculated using a standard curve that was plotted as absorbance versus Collagen Iα, Collagen III, and TGF‐β1 concentration. All samples and standards were measured in duplicate.

### 2.13 Data acquisition and re‐analysis

Human TGF‐β expression levels in different tumour types from TCGA database compared to adjacent normal tissues were analysed and visualized in Tumour Immune Estimation Resource (TIMER; https://cistrome.shinyapps.io/timer/).^28^ Association between expression levels of TGF‐β and EMT markers was estimated in TIMER. Types and frequency of human TGF‐β in different types of breast cancer patients were using a pre‐existing cancer gene expression database in cBioPortal (https://www.cbioportal.org). The correlation between TGF‐β and breast cancer patients' DFS was analysed using the Kaplan–Meier Plotter^29^ provided in Breast Cancer Gene‐Expression Miner v4.8.^30^

### 2.14 Xenograft tumour mouse model

Eight‐week‐old male BALB/c nude mice were purchased from the animal centre of Guizhou Medical University. The mice were housed in pathogen‐free barrier facility with a 12 h light/dark cycle and fed ad libitum with sterile feed and water. All animal experimental procedures were approved and conducted by the Committee for Animal Experiments, Guizhou Medical University. The mice were allowed for an acclimation to the housing environment for 7 days after arriving in the facility. To evaluate in vivo anti‐tumour efficacy, MDA‐MB‐231 cells (10^6^ cells/mouse) were injected into the left subaxillary of the mice. The recipient mice were then randomly divided into three groups (*N* = 3 mice per group) receiving daily systemic injections with PFD (200 mg/kg), or once every 3 days systemic injections with nab‐PTX (10 mg/kg) or the combination of the two drugs, respectively, for 36 days. The tumour cell‐injected mice giving no treatment was used as controls. Body weight and tumour volume (0.5 × Length × Width^2^)^31^ of all the experimental mice were recorded every 4 days, while the tumour size and weight at endpoint were measured when the tumours were excised.

### 2.15 Statistical analysis

Data are presented as the mean ± SD of three independent experiments. Statistical differences between treatment and control groups were via one‐way anova test using Prism GraphPad Prism software. A *p* Value less than 0.05 was considered statistically significant.

## 3 RESULTS

### 3.1 TGF‐β expression is increased in breast cancer and negatively associated with DFS

**FIGURE 1:**
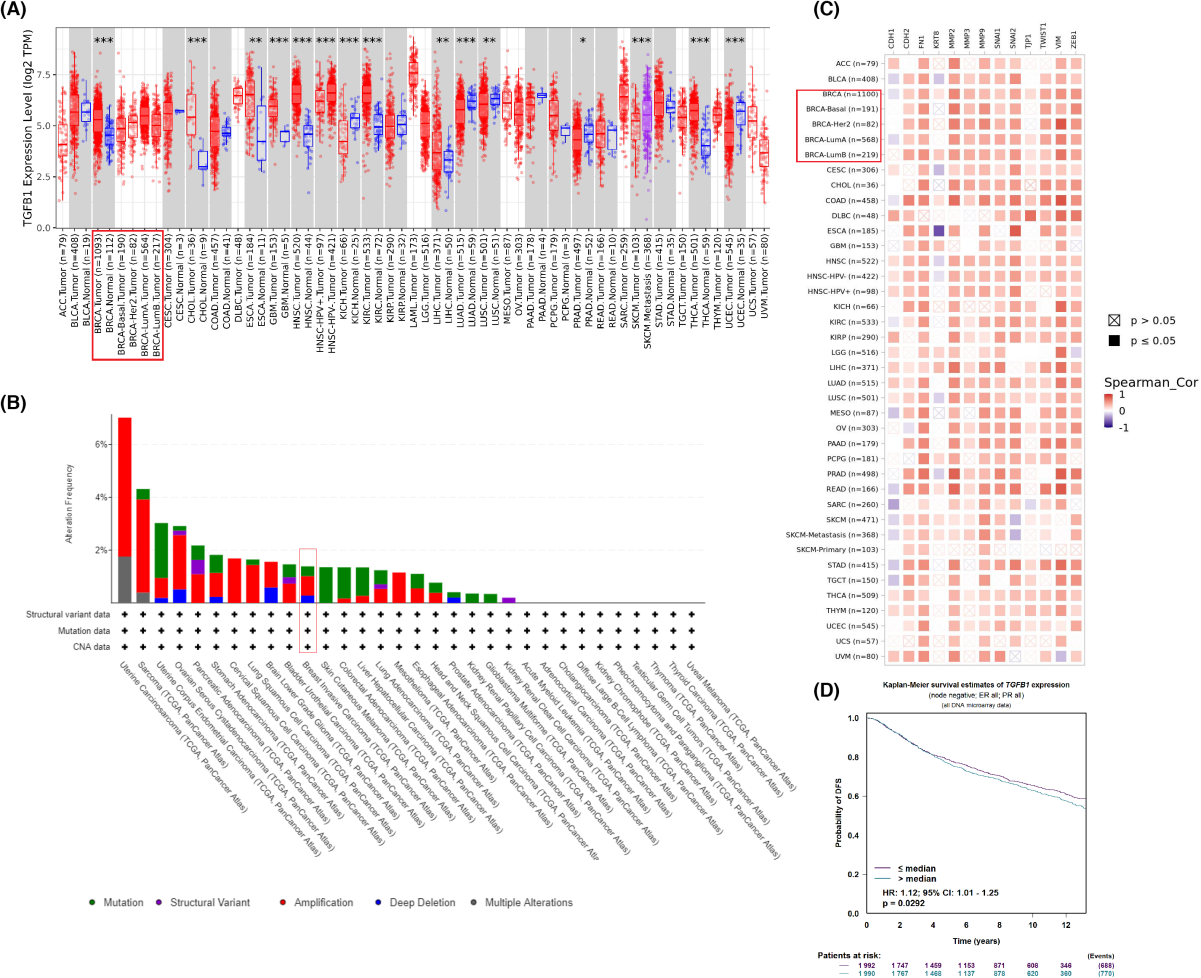
Expression of TGF‐β and EMT‐associated genes in breast cancer tissues. (A) Pan‐cancer expression profile of TGF‐β in TCGA cohorts. Breast cancer (BRCA) subtypes were highlighted with red rectangle. (B) The genetic alteration type and frequency of TGF‐β in different cancer tissues. Breast invasive carcinoma was highlighted with red rectangle. (C) Heatmap showing Spearman's correlations between mRNA expression of EMT‐related genes and metastasis across Pan‐cancer. BRCA type was highlighted with red rectangle. (D) Evaluation of the correlation of TGF‐β expression with DFS of breast invasive cancer by Kaplan–Meier method.

Metastasis or stage IV cancer is at a stage when tumour cells spread from the original site to surrounding and distant parts of the body. Metastasis is estimated to account for approximately 90% of cancer deaths.^32^ One of the well acknowledged causes of metastasis is EMT through which cancer cells acquire stem cell‐like properties and exhibit enhanced apoptosis and chemotherapeutic resistances.^27^, ^33^, ^34^ As TGF‐β is known as a potent EMT driver,^21^ we confirmed the association of expression of TGF‐β and EMT‐related genes with breast cancer carcinogenesis and metastasis in TCGA pan‐cancer dataset. Indeed, TGF‐β expression level is elevated in overall breast cancer tissues in comparison to the corresponding normal control (Figure 1A). More importantly, amplification of TGF‐β gene is the most frequent type of mutation associated with breast invasive carcinoma (Figure 1B), which is in keeping with the fact that TGF‐β is a strong inducer of EMT.^21^ We also observed a close association between metastasis and the increased expression of numerous EMT‐related genes across pan‐cancer, including different subtypes of breast invasive carcinomas (Figure 1C). Finally, an estimation of the effect of TGF‐β expression on DFS of overall breast cancer patients by Kaplan–Meier method showed that a TGF‐β mRNA expression level over median level is significantly (*p* < 0.05) associated with more reduced DFS than the patients with lower level of TGF‐β (Figure 1D).

### 3.2 PFD inhibits MDA‐MB‐231 cells proliferation, migration, and promotes their apoptosis in vitro

**FIGURE 2:**
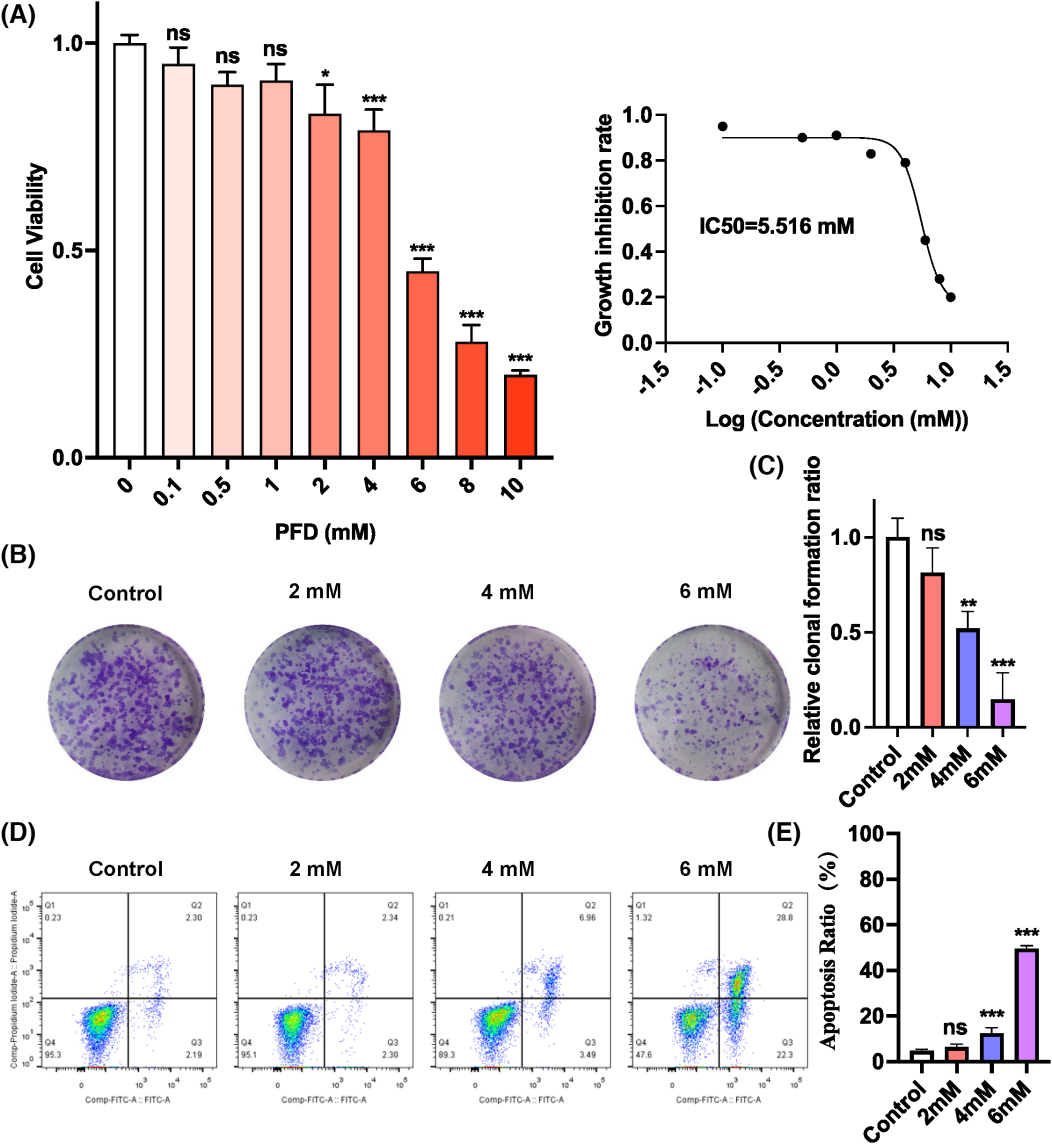
Effects of PFD on proliferation, colony formation and apoptosis in MDA‐MAB‐231 cells. (A) Cell viability of PFD‐treated MDA‐MB‐231 cells. The IC50 value was calculated based on CCK‐8 assay results after 24 h treatment. (B, C) Clonogenic assay. Representative images (B) and quantification (C) of the colony‐forming ability of MDA‐MB‐231 in the absence or presence of PFD for 24 h. (D, E) Apoptosis in PFD‐treated MDA‐MB‐231 cells determined by Annexin V/PI double staining assay. Representative flow cytometry plots (D) and quantification (E) of apoptotic cells after 24 h treatment of gradient PFD. *p < 0.05, **p < 0.01, ***p < 0.001 versus control. ns, non‐significant.

**FIGURE 3:**
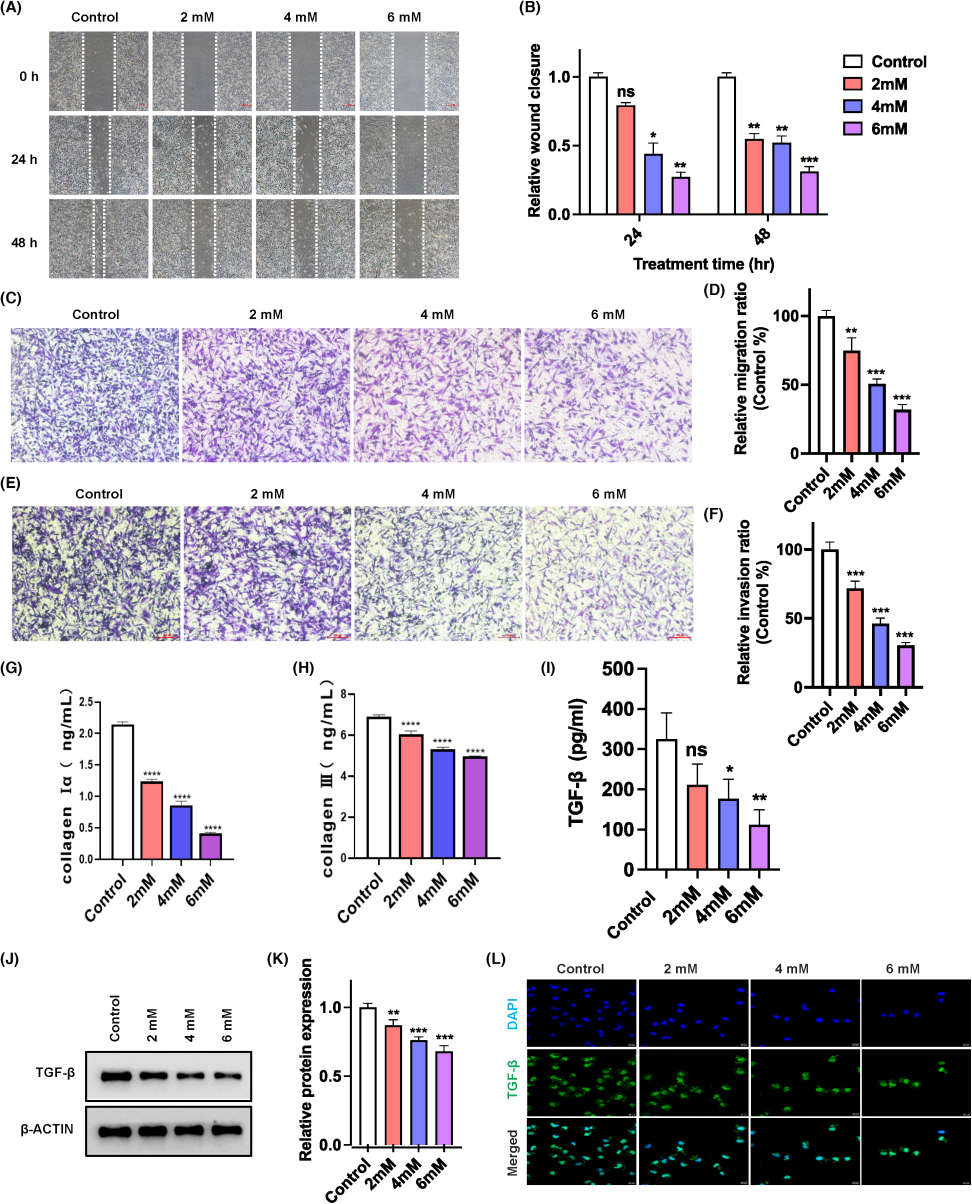
Effects of PFD on migration/invasion and TGF‐β production in MDA‐MAB‐231 cells. (A, B) Wound healing assay. Representative images (A) and quantification (B) of migrating capacity of MDA‐MB‐231 in the absence or presence of PFD for 24 h. (C, D) Transwell migration assay. Representative images (C) and quantification (D) of MDA‐MB‐231 cells migrated through the upper transwell chamber in the absence or presence of PFD. (E, F) Transwell invasion assay. Representative images (E) and quantification (F) of MDA‐MB‐231 cells migrated through the matrigel‐coated upper transwell chamber in the absence or presence of PFD. (G) Measurement of CollagenIα content in MDA‐MB‐231 culturing medium by ELISA. (H) Measurement of Collagen III content in MDA‐MB‐231 culturing medium by ELISA. (I) Measurement of TGF‐β content in MDA‐MB‐231 culturing medium by ELISA. (J, K) TGF‐β expression determined by western blot assay. Representative images (D) and quantification (E) of the intracellular TGF‐β expression in PFD‐treated MDA‐MB‐231 cells. β‐ACTIN was used as an internal control of equal total protein loading. (L) Representative immunofluorescence images showing suppression of TGF‐β expression in PFD‐treated MDA‐MB‐231 cells. DAPI was included as a nuclear counterstain. *p < 0.05, **p < 0.01, ***p < 0.001 versus control. ns, non‐significant.

PFD is an antifibrotic drug for treatment of idiopathic pulmonary fibrosis.^18^ Previous study demonstrated that it exhibits a strong inhibitory effect on TNBC tumour growth induced by cancer‐associated fibroblasts (CAFs).^35^ At the beginning of this study, we asked whether PFD has anti‐tumour effect by directly targeting cancer cells. To address the question, we firstly determined the anti‐tumour effects of PFD in MDA‐MB‐231, a type of highly aggressive, invasive, and poorly differentiated TNBC cells. As shown in Figure 1, PFD exerted a dose‐dependent inhibition on cell proliferation, with the half maximal inhibitory concentration (IC50) of about 5.516 mM (Figure 2A). The cytotoxic effect of PFD on MDA‐MB‐231 cells was further indicated by the dosage‐dependent decrease in colony formation observed in clonogenic assay (Figure 2B,C) and significantly induced cell death detected by Annexin V/PI apoptosis assay (Figure 2D,E). Moreover, the migrating/invasive capacity of MDA‐MB‐231 cells was also significantly attenuated by PFD as indicated by the wound healing and transwell assays. Specifically, in wound healing assay, MDA‐MB‐231 cells migrated only 0.79 (*p* = 0.0523), 0.44 (*p* = 0.0112), and 0.27 (*p* = 0.0036) times the distance of untreated cells in the presence of 2‐, 4‐ and 6‐mM PFD, respectively, for 24 h (Figure 3A,B). Such concentration‐dependent “hard‐to‐heal” scratches were remained until 48 h after the drug treatment when the scratch was getting to closure in the untreated cells (Figure 3A,B). In transwell assay, only 74.8% (*p* = 0.0017), 50.7% (*p* < 0.001) and 31.9% (*p* < 0.001) of MDA‐MB‐231 cells were able to migrate from the upper transwell chamber to the lower chamber containing the complete growth medium upon treatment with 2‐, 4‐ and 6‐mM PFD, respectively (Figure 3C,D). Similarly, the invasive capacity was also compromised by PFD as indicated by a 28.2% (*p* = 0.0002), 53.8% (*p* < 0.001) and 69.4% (*p* < 0.001) reduction in the number of invaded cells through the Matrigel‐coated upper insert chamber to the lower chamber in the presence of increasing dosages of PFD, respectively (Figure 3E,F). Taken together, these data clearly showed that the antifibrotic drug PFD is cytostatic and pro‐apoptotic to breast cancer cells.

It was reported that MDA‐MB‐231 cells rely on autocrine TGF‐β signalling for positive regulation of cell growth.^36^ A significant increase in apoptosis was also observed in MCF‐7 cells with the abrogated autocrine TGF‐β signalling.^37^ In addition, evidence from a previous study showed that PFD attenuates TGF‐β‐induced fibroblast activity and restores fibroblast‐mediated collagen gel contraction and migration.^19^ Therefore, we examined the effect of PFD on Collagen Iα and Collagen III production in breast cancer cells. We found that the generation of CollagenIα and Collagen III was significantly reduced after PFD administration (Figure. 3G,H). At the same time, we also tested the effect of PFD on TGF‐β production in these breast cancer cells. Strikingly, PFD exhibited a dose‐dependent inhibition on the autocrine TGF‐β production in the cultured MDA‐MB‐231 cells (Figure 3I). The decreased expression of TGF‐β protein in the presence of PFD was further confirmed by western blot (Figure 3J,K) and immunofluorescence (Figure 3L) assays.

### 3.3 PFD represses TGF‐β‐induced EMT in breast cancer cells

**FIGURE 4:**
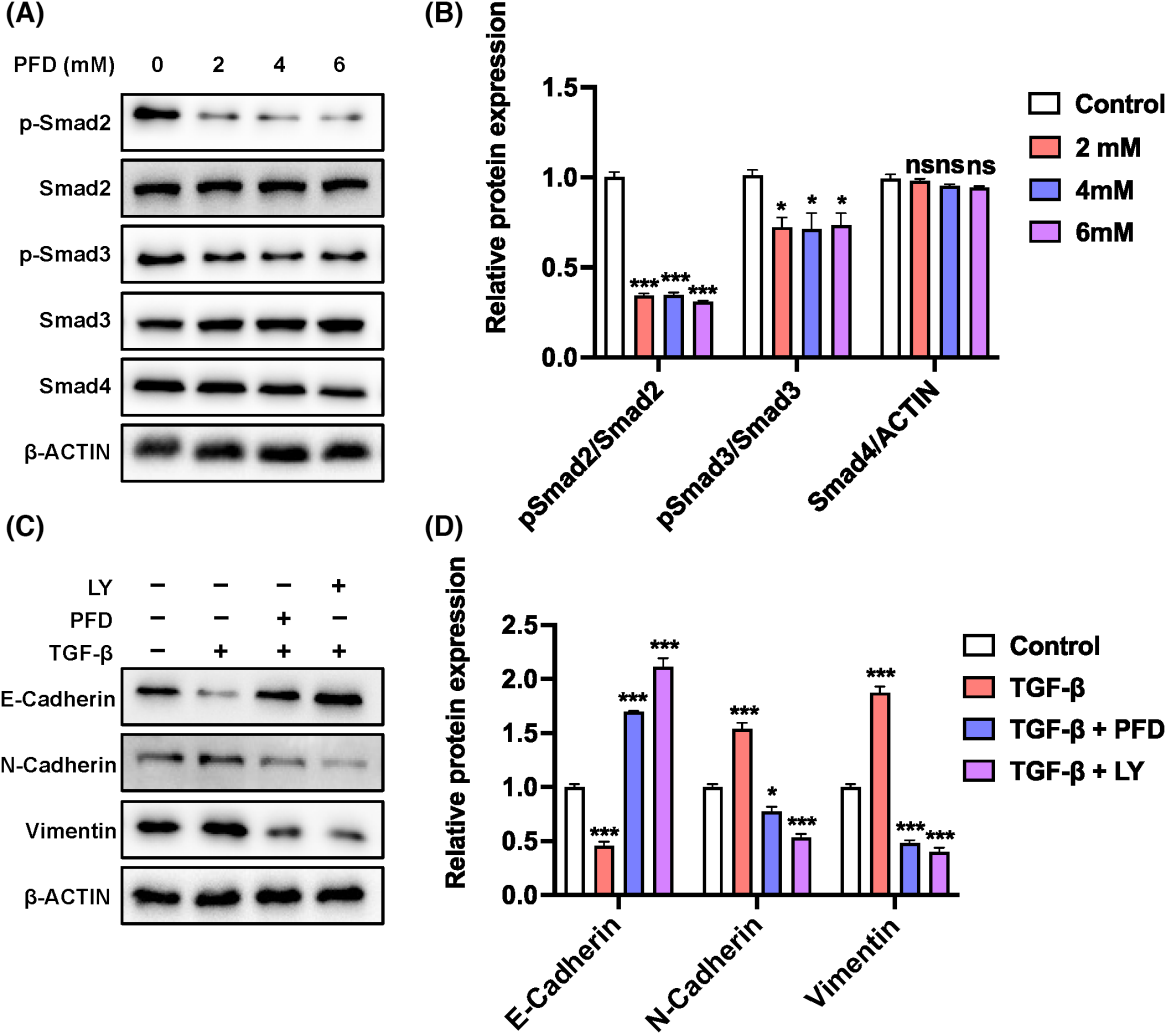
Effects of PFD on activation of Smad signalling pathway and protein expression of EMT‐related genes in TGF‐β‐treated MDA‐MAB‐231 cells. (A, B) The effect of PFD on TGF‐β‐stimulated Smad signalling pathway. Representative western blot images (A) and quantification (B) of phosphorylated and total Smad family proteins. (C, D) The effect of PFD on TGF‐β‐induced EMT. Representative western blot images (C) and quantification (D) of epithelial marker E‐Cadherin (E‐Cad) and mesenchymal genes such as Vimentin (VIM) and N‐Cadherin (N‐Cad). In all Figure 4 ‐related experiments, MBA‐MB‐231 cells were pre‐treated with TGF‐β (5 ng/ml) for 24 h followed by exposure to gradient PFD or the selective TGF‐β receptor type I/II dual inhibitor LY2109761 (LY, 2 μM) for another 24 h. *p < 0.05, **p < 0.01, ***p < 0.001 versus control. ns, non‐significant.

Activation of Smad signalling is essential in mediating TGF‐β‐induced EMT.^21^ Therefore, we further examined the effects of PFD on TGF‐β‐stimulated activation of Smad signalling pathway. Strikingly, PFD inhibited activation of Smad signalling pathway by TGF‐β as indicated by the decreased phosphorylation of Smad2, and, to a less extent, Smad3, whereases the expression of Smad4 was undisturbed throughout the tested doses of PFD (Figure 4A,B). Accordingly, PFD strongly prohibited TGF‐β‐induced expression of mesenchymal marker genes, including N‐Cadherin and VIMENTIN (VIM) (Figure 4C,D). Instead, the TGF‐β‐suppressed epithelial signature, E‐Cadherin, was restored upon addition of PFD (Figure 4C,D). These mirrored the effects of LY2109761 which is a TGF‐β receptor type‐I and type‐II dual inhibitor^38^ (Figure 4C,D), suggesting that PFD might suppress metastasis through disturbing TGF‐β‐induced EMT in breast cancer cells.

**FIGURE 5:**
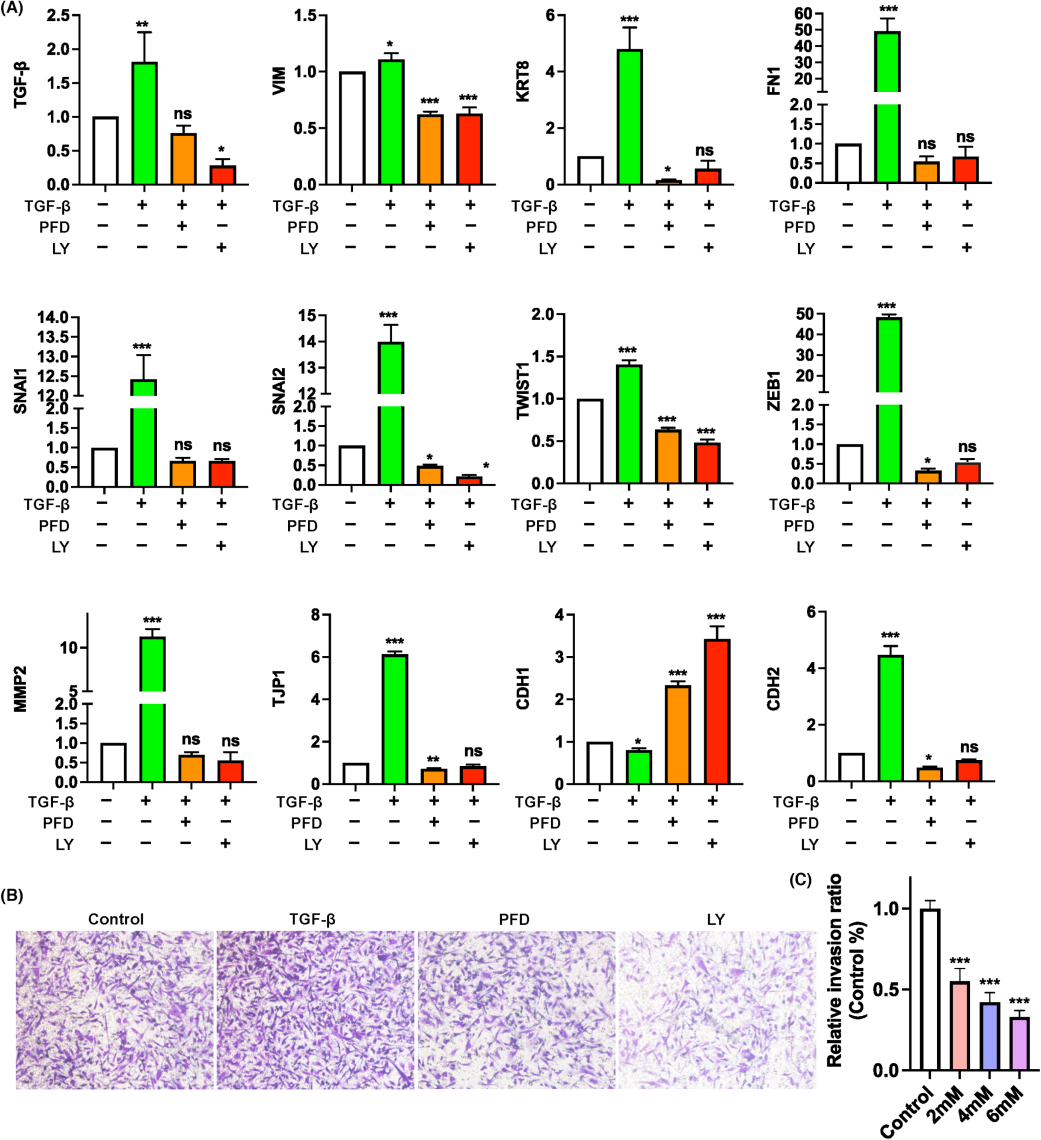
Effects of PFD on EMT‐related gene expression and invasion of MDA‐MAB‐231 cells. (A) qPCR determining expression of TGF‐β and EMT‐related genes. MBA‐MB‐231 cells were pre‐treated with TGF‐β (5 ng/ml) for 24 h followed by exposure to gradient PFD or LY2109761 (LY, 2 μM) for another 24 h. (B, C) Transwell invasion assay. Representative images (B) and quantification (C) of MDA‐MBA‐231 cells invaded through Matrigel‐coated upper transwell chamber. *p < 0.05, **p < 0.01, ***p < 0.001 versus control. ns, non‐significant.

By quantitative real‐time PCR, we further demonstrated that TGF‐β induced the expression of numerous downstream EMT‐associated genes, including *SNAI2*, *TWIST1*, Zinc finger E‐box‐binding homeobox 1 (*ZEB1*), Keratin 8 (*KRT8*), Fibronectin 1 (*FN1*), and Matrix metalloproteinase 2 (*MMP2*) as well as mesenchymal genes such as *VIM* and CDH2/N‐Cadherin, which were, however, significantly down‐regulated by PFD (Figure 5A). The epithelial gene CDH1/E‐Cadherin which is commonly lost during TGF‐β‐induce EMT was significantly up‐regulated instead, in the presence of PFD (Figure 5A). Indeed, as observed in the transwell assay, although the exogenous TGF‐β strengthened largely the invasion capacity of MDA‐MB‐231 cells, the number of the invaded cells was significantly reduced upon supplementation of either PFD or LY2109761 (Figure 5B,C).

### 3.4 PFD inhibits xenografted tumour growth in vivo

**FIGURE 6:**
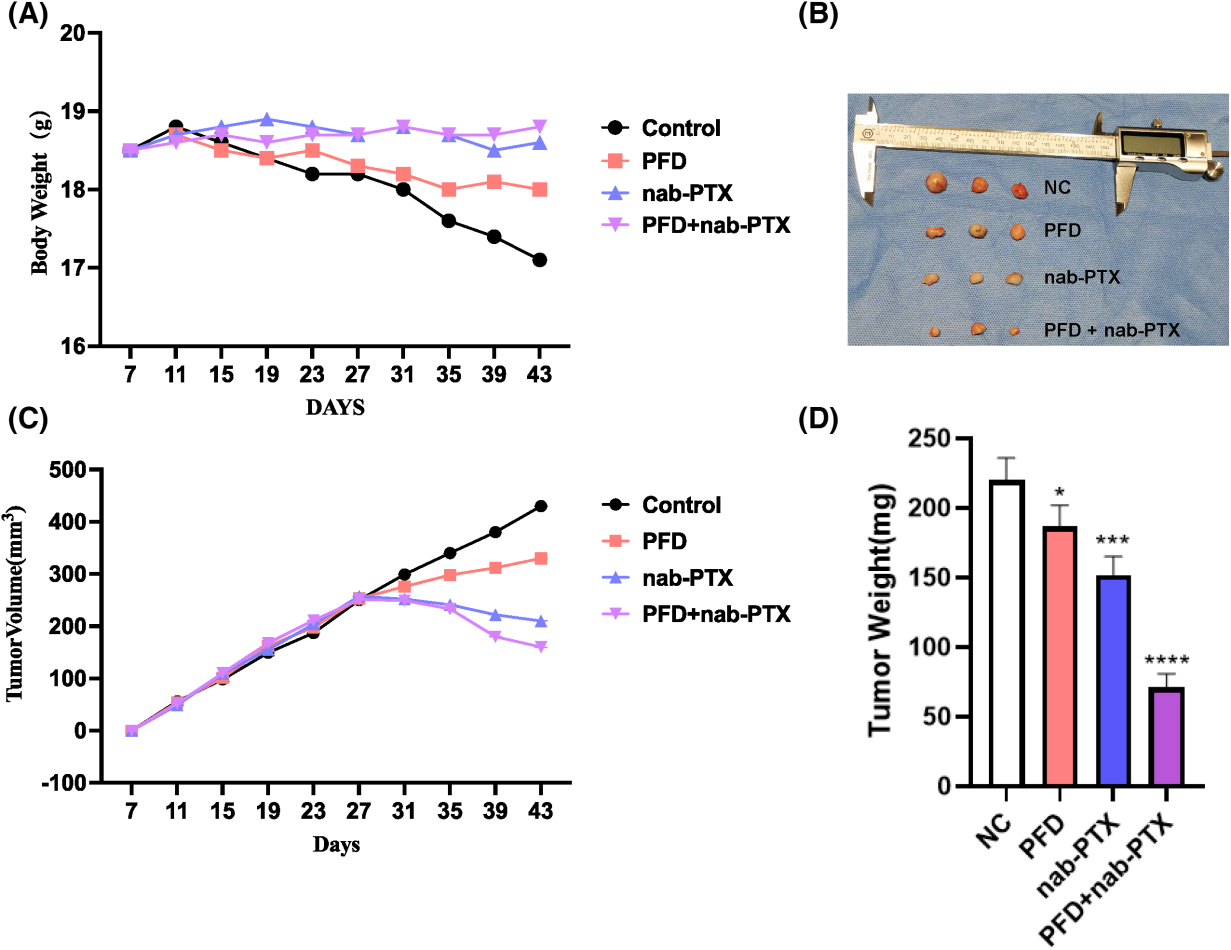
Effects of PFD on breast tumour growth in xenograft mouse model. (A) Body weight growth curve of xenograft tumour‐bearing mice. (B–D) Tumour size (B), volume (C), and weight (D) in xenograft mice. Immunosuppressive nude mice were acclimated to housing environment for 7 days followed by a subaxillary injection of 10^6^ MDA‐MB‐231 cells. The mice were then randomly sub‐grouped for systemic treatments by PFD (200 mg/kg), nab‐PTX (10 mg/kg) or a combination of the two drugs, respectively, for 36 days. The tumour cell‐injected mice receiving no treatment were used as Control. *p < 0.05, **p < 0.01, ***p < 0.001 versus Control.

**FIGURE 7:**
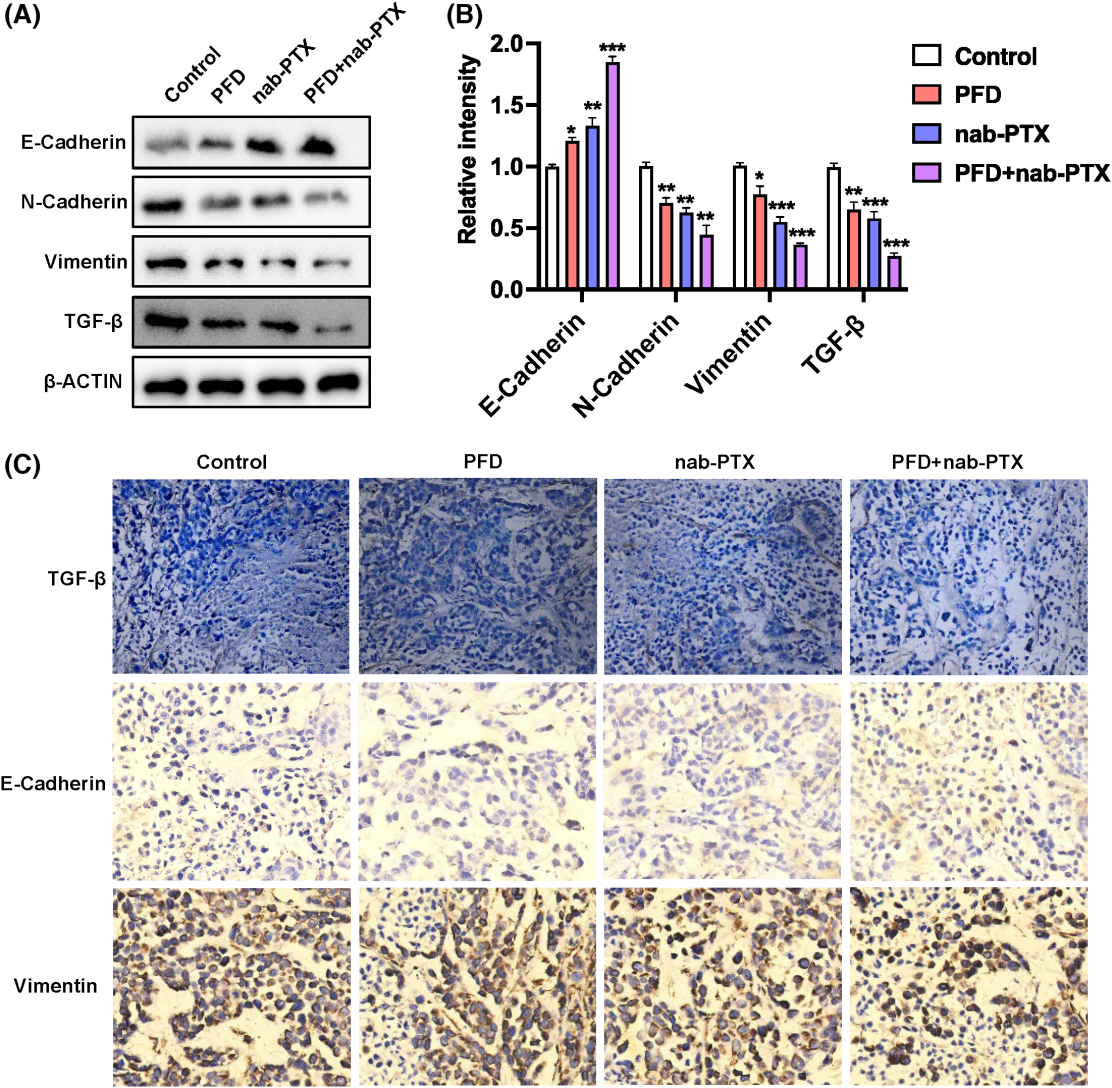
Effects of PFD on the expression of TGF‐β and EMT marker proteins in in vivo xenograft tumours. (A, B) Representative western blot images (A) and quantification (B) of epithelial marker (E‐Cadherin, E‐Cad) and mesenchymal genes such as VIM and N‐Cadherin (N‐Cad). (C) Representative immunohistochemistry showing suppression of TGF‐β expression and EMT in primary tumours from PFD‐, nab‐PTX‐ or combinatory drug‐treated recipient mice.

We continued to evaluate the anti‐tumour effect of PFD in vivo. Immunocompromised BALB/c nude mice were given a subaxillary injection of MDA‐MB‐231 cells after seven‐day acclimation to the housing environment. On the same day, the mice were treated systemically with PFD, nanoparticle albumin‐bound paclitaxel (nab‐PTX), an FDA‐approved chemotherapy drug for breast cancer,^39^ as well as a combination of the two, respectively. It is known that cancer causes cachexia which is a complicated metabolic syndrome accompanied with progressive loss of body weight.^40^, ^41^ To evaluate such tumour burden under different treatment conditions, we continuously monitored the variation in body weight of the experimental mice during the following 1 month after the treatment. As expected, the tumour‐bearing mice with no treatment lost nearly 10% of their body weight (Figure 5A) and developed the largest tumour (Figure 5B–D) by the end of the experiment. On the contrary, the mice receiving either nab‐PTX or the combinatory regimen were able to keep body weight stable throughout the experiment, while the mice receiving PFD treatment alone reduced approximately 5% of their body weight (Figure 6A). Correspondingly, PFD significantly augmented the anti‐tumour effect of nab‐PTX as indicated by the observation that tumours in the combinatory drugs‐treated group were smallest in both size (Figure 6B,C) and weight (Figure 6D). Although significant, PFD alone only exhibited a moderate inhibition on the xenograft tumour grow in vivo (Figure 6B–D). Notably, we detected high levels of TGF‐β, N‐Cadherin, and VIM proteins but low level of E‐Cadherin in the xenograft tumours of control group (Figure 7A,B). The expression of TGF‐β and the mesenchymal marker proteins such as N‐Cadherin and VIM in the xenograft tumours were significantly reduced in all treatment regimen groups as determined by both western blot (Figure 7A,B) and immunohistochemistry (Figure 7C). Instead, the epithelial signature protein E‐Cadherin was significantly induced in all three treated tumours with the highest expression being observed in the PFD and nab‐PTX combinatory treatment group (Figure 7A–D).

**FIGURE 8:**
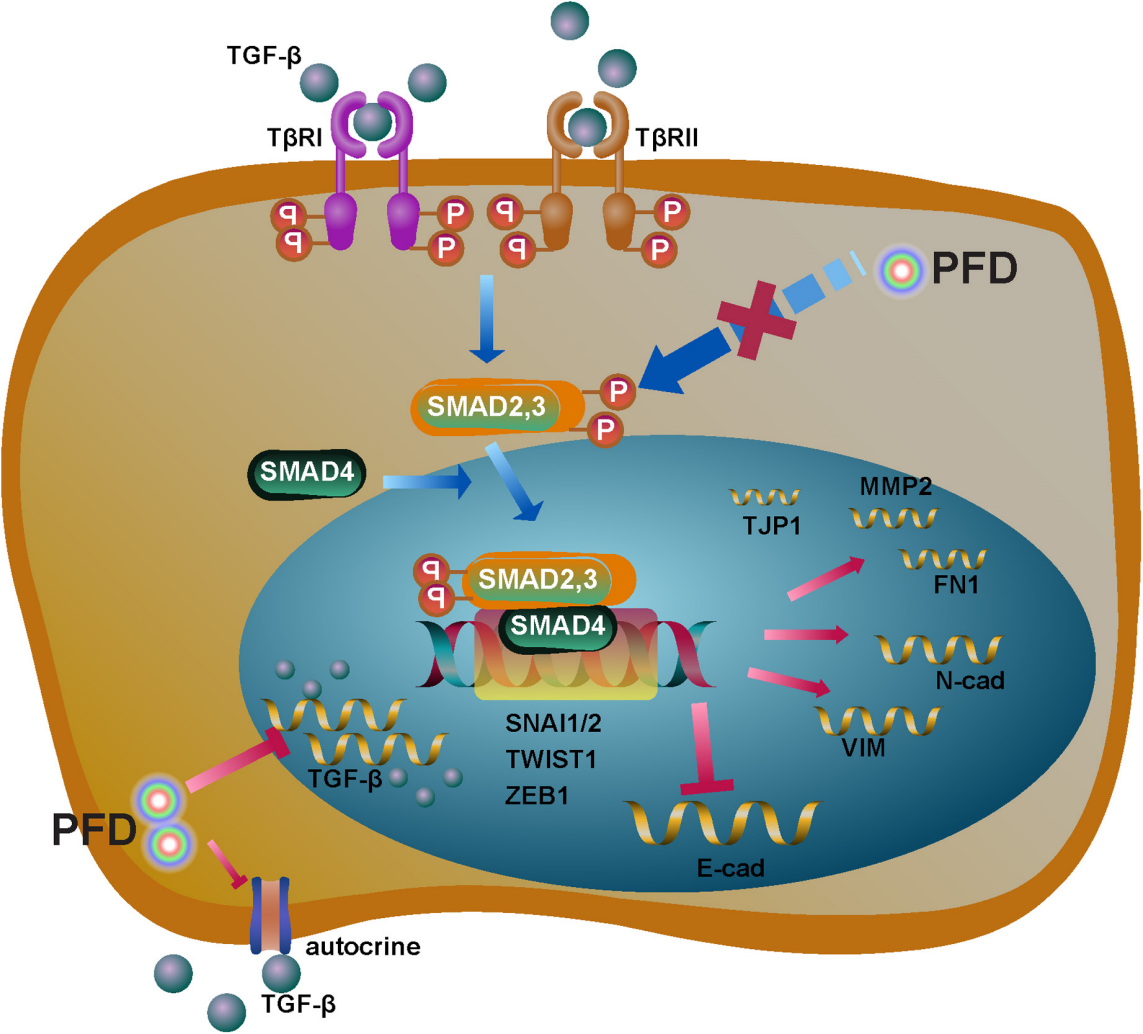
Mechanistic diagram of PFD actions on TGF‐β‐induced EMT program TGF‐β binds to a tetrameric complex of TβRI and TβRII on cell membrane, which consequently phosphorylates Smad2 and Smad3 proteins. Smad2 and Smad3 proteins are then dimerized and combined further with Smad4. The resultant heterotrimer translocates into the nucleus and activates expression of EMT‐inducing transcription factors, including SNAI2, ZEB1, TWIST1, etc. PFD strongly suppresses TGF‐β‐induced phosphorylation of Smad‐2 and ‐3 and thereby inhibits the downstream EMT program. Meanwhile, PFD also represses TGF‐β transcription and thus reduces the autocrine TGF‐β in breast cancer cells. The elements of the diagram were adopted from library of icons in Reactome.^52^

Taken together, TGF‐β is a chief culprit leading to breast cancer metastasis through inducing EMT in tumour cells. As summarized in Figure 8, PFD acts as a double‐edge sword in preventing breast cancer metastasis. It suppresses both transcription and autocrine production of TGF‐β in tumour cells. In the meantime, PFD also impairs TGF‐β‐stimulated phosphorylation on Smad2 and Smad3 proteins, which consequently leading to down‐regulation of transcription factors, including SNAI2, ZEB1, and TWIST1 and thus EMT reprogramming.

## 4 DISCUSSION

Although treatable, breast cancer is difficult to cure when it has spread to surrounding or remote parts of the body, such as bones, lungs, brain, or liver. A recent statistic from the American Cancer Society estimates that the five‐year survival rate of metastatic breast cancer is 29% for women.^1^ Recently, tumour fibrosis and inflammation have been increasingly recognized as important factors which influence tumour progression and metastasis. Over‐expression of TGF‐β ligands in cancers at late stage has been reported to be positively associated with the aggressive and metastatic phenotype.^42^, ^43^ We noticed that one of the mechanisms of the anti‐fibrotic drug, Pirfenidone, for treating idiopathic pulmonary fibrosis is its inhibitory effect on TGF‐β signalling pathway.^44^ Accordingly, we hypothesized that it might be used for counteracting the pro‐progression role of TGF‐β in breast cancer. Indeed, evidence from the earlier studies shows that PFD enables normalization of tumour extracellular matrix by reducing collagen and hyaluronan tumour components, through which it improves delivery and efficacy of doxorubicin in xenograft breast cancer.^45^ Concomitantly, the synergistical effect of PFD on the anti‐tumour efficacy of doxorubicin is also via depletion of CAFs.^35^ Notably, data from these studies demonstrated that although PFD synergistically inhibited primary tumour growth and lung metastasis in combination with doxorubicin in TNBC xenograft mouse model, little or no effect by this anti‐fibrotic drug monotherapy was observed on primary tumour growth and lung metastasis.^35^ We think it could because of the relatively lower concentrations of the drug (100 μM in vitro and 50 mg/kg in vivo) that were used in those studies. Our data showed that the IC50 value of PFD in MDA‐MB‐231 cells is 5.516 mM (Figure 2A). Administer of the drug at dosages close to the IC50 value exhibited significant growth and migration/invasion inhibitory as well as pro‐apoptotic effects on MDA‐MB‐231 cells. Importantly, according to a previous in vitro study in rat normal hepatic stellate cells, the millimolar level of PFD does not affect cell viability.^46^ Therefore, future study assessing the tolerance range of PFD to normal mammary gland epithelial cells will facilitate application of PFD in breast cancers therapy at advanced stage.

During tumour progression, cell proliferation and fibrosis take into account the stiffness and aggressiveness of the tumour as well as lymphatic invasion. While EMT or fibrosis or EMT combined with fibrosis appears to contribute to tumour metastasis, the exact contribution is unknown. Solid tumours are highly heterogeneous and consist of not only cancer cells but also many other TME resident cells, including fibroblasts, endothelial cells, epithelial cells, and infiltrating immune cells. One of the novel aspects of our current study is that unlike the existing studies aiming on the role of PFD in non‐cancer resident cells, e.g., suppressing collagen production by CAFs,^35^, ^45^ we had focused on exploring its direct actions on tumour cells. It is well known that TGF‐β is overproduced in almost all the advance human tumours and is positively associated with tumour growth, invasion, and metastasis.^47^, ^48^ The autocrine TGF‐β signalling has been demonstrated as essential in promoting survival of MCF‐7 cells but not untransformed human mammary epithelial cells through activating Erk but inhibiting p38 signalling pathways.^37^ Our data showed that PFD treatment represses autocrine TGF‐β production in MDA‐MAB‐231 cells in vitro, leading to a dose‐dependent growth inhibition and apoptosis. However, under our experimental conditions, PFD alone did not exert strong anti‐tumour effect in vivo although it did significantly ameliorate tumour burden‐caused body weight loss and inhibit xenograft tumour growth to a less extent than the chemotherapy drug nab‐PTX did. It would be interesting to examine the long‐term efficacy of PFD administration on tumour growth inhibition or metastasis prevention. It has been shown that pirfenidone inhibits fibrotic progression in part by inhibiting TGF‐β, which is preferentially activated in fibrotic tissues. Combining drugs may result in better therapeutic efficacy and reduce the likelihood of cancer cells developing drug resistance as a result of the synergistic action of multiple drugs.

Nevertheless, we are excited to see PFD synergistically boosted cancer‐killing effect of nab‐PTX in vivo. This could be partially due to its strong repression on TGF‐β expression as well as TGF‐β‐induced EMT. TGF‐β is known as a potent inducer of EMT which is a driving force of cancer metastasis.^33^ Mechanistically, TGF‐β induces EMT through phosphorylating Smad2 and Smad3.^49^, ^50^ The activated Smads induce subsequently the expression of transcriptional repressors, including SNAI2, ZEB1/2 and TWIST etc.^21^ These are well‐established E‐Cadherin repressors through inducing hypermethylation and histone deacetylation at the promoter.^51^ Along with the loss of E‐Cadherin, the mesenchymal phenotype signature genes such as N‐Cadherin and VIM are up‐regulated instead. Such epithelial phenotype to mesenchymal phenotype conversion liberates the tight adherent junction‐confined tumour cells and facilitates their free migration to the surrounding tissues.^33^ We saw TGF‐β‐activated Smad signalling pathway and most of the EMT‐inducing transcription factors as well as the epithelial and mesenchymal signature genes were significantly suppressed or reversed by PFD, which was a more pronounced phenotype than direct killing of tumour cells.

## AUTHOR CONTRIBUTIONS

**qin dai Luo:** Conceptualization (equal); data curation (equal); writing – original draft (equal). **lin xian Zeng:** Data curation (equal); formal analysis (equal). **ling shu Zhang:** Methodology (equal); resources (equal). **hong dao Li:** Investigation (equal); methodology (equal). **mei zhi Cheng:** Investigation (equal); methodology (equal). **yun Wang:** Project administration (equal); resources (equal). **hua jin Long:** Project administration (equal); supervision (equal). **quan zu Hu:** Funding acquisition (equal); project administration (equal); supervision (equal). **qi shi Long:** Investigation (equal); resources (equal). **jing zhou:** Methodology (equal); resources (equal). **suai Zhang:** Supervision (equal); validation (equal); writing – review and editing (equal). **zhu Zeng:** Project administration (equal); resources (equal); supervision (equal); writing – review and editing (equal).

## CONFLICT OF INTEREST

The authors declare no conflict of interest.

## Data Availability

The data that support the findings of this study are available from the corresponding author upon reasonable request.
